# Effect of Handwriting on Visual Word Recognition in Chinese Bilingual Children and Adults

**DOI:** 10.3389/fpsyg.2021.628160

**Published:** 2021-05-28

**Authors:** Connie Qun Guan, Elaine R. Smolen, Wanjin Meng, James R. Booth

**Affiliations:** ^1^Faculty of Foreign Studies, Beijing Language and Culture University, Beijing, China; ^2^Department of Psychology, Carnegie Mellon University, Pittsburgh, PA, United States; ^3^Teachers College, Columbia University, New York City, NY, United States; ^4^Institute of Psychology, Moral and Special Education, National Institute for Education Sciences, Beijing, China; ^5^Department of Psychology and Human Development, Vanderbilt University, Nashville, TN, United States

**Keywords:** handwriting, embodied cognition, N170, laterality, plasticity

## Abstract

In a digital era that neglects handwriting, the current study is significant because it examines the mechanisms underlying this process. We recruited 9- to 10-year-old Chinese children (*n* = 24), who were at an important period of handwriting development, and adult college students (*n* = 24), for both behavioral and electroencephalogram (EEG) experiments. We designed four learning conditions: handwriting Chinese (HC), viewing Chinese (VC), drawing shapes followed by Chinese recognition (DC), and drawing shapes followed by English recognition (DE). Both behavioral and EEG results showed that HC facilitated visual word recognition compared to VC, and behavioral results showed that HC facilitated visual word recognition compared to drawing shapes. HC and VC resulted in a lateralization of the N170 in adults, but not in children. Taken together, the results of the study suggest benefits of handwriting on the neural processing and behavioral performance in response to Chinese characters. The study results argue for maintaining handwriting practices to promote the perception of visual word forms in the digital age.

## Introduction

The development of the ability to write meaningful symbols was a major milestone in the development of human civilization. Handwriting serves to link auditory and motor routines with visual word processing, which is a hallmark for successful reading (Dehaene and Cohen, 2011). Early processing of visual word forms is constrained by the interaction with auditory and motor regions (Sekiyama et al., 2003; Wuerger et al., 2012; Callan et al., 2014), and the mechanism elicited by handwriting movement facilitates the auditory and motor integration of visual word forms (Longcamp et al., 2006; Guan et al., 2011; James, 2017).

Handwriting using Chinese characters appears to differ in several important ways from writing using an alphabetic system, such as that used in English. When handwriting Chinese, the individual needs to extract the visual–spatial features of the characters first. In contrast, for alphabetic words, phonological processing, such as mapping the letters corresponding to the phonemes, is more important. Giving up handwriting may affect how future generations learn to read (James and Engelhardt, 2012; Tan et al., 2013). Reducing handwriting instruction and practice may contribute significantly to difficulties in children’s reading development (James, 2010; Guan et al., 2011; Tan et al., 2013) and overall writing skills (Daly et al., 2003; van Reybroeck and Michiels, 2018; Guan et al., 2019) in Chinese and Western languages.

Handwriting affects symbol learning by creating a network that includes both sensory and motor brain systems. James (2017) have demonstrated that the motor system creates variability (through handwriting in this case) in our perceptual world that enhances behavioral performance and serves to link brain systems into functional networks. In addition, a series of handwriting behavioral studies in both native English-speaking adults and Chinese beginning readers has suggested that handwriting Chinese characters focuses attention on stroke components (Guan et al., 2015) and facilitates orthographic recognition to aid reading acquisition among Chinese learners (Guan and Fraundorf, 2020; Guan et al., 2020). It may even be the case that drawing promotes Chinese children’s cognitive ability in reading Chinese characters (Tan et al., 2013). A practical implication of these studies is that handwriting practice can be important parts of courses in Chinese to support more robust student learning of the spoken and written language.

The N170 is a component of the event-related potential (ERP) and is a neurophysiological indicator of early visual word recognition. Visual specialization for reading is revealed by the topography of the N170 ERP response (Maurer et al., 2005a). The N170 ERPs seem to represent a logographic processing strategy in visual word recognition (Simon et al., 2007). Selectivity of the N170 in the left hemisphere is also an electrophysiological marker for expertise in reading Chinese (Zhao et al., 2012) and Japanese (Maurer et al., 2008). However, whether handwriting experience enhances the N170 is unknown. We did not focus on other early visual ERP indicators (such as P1 and N1) because they are non-linguistic (Planton et al., 2013; Rothe et al., 2015). Focusing only on N170 modulation and the laterality effect is innovative, as previous relevant studies did not manipulate handwriting experience. Therefore, whether handwriting experience compared to other learning conditions might trigger this N170 modulation is unknown.

In summary, there is still controversy to what extent handwriting can promote the perception of words/characters. In particular, whether handwriting Chinese might promote visual word recognition more than visual perception or drawing is still unexplored. Moreover, there have been no direct studies comparing the role of handwriting in learning for children vs. adults.

### The Current Study

The current study focuses on not only the difference between handwriting and viewing but also the difference between handwriting and drawing followed by Chinese recognition and drawing followed by English recognition. Specifically, we investigate whether the early neural mechanism of visual processing is different between the four learning conditions by examining the N170. The following research questions guide the present investigation:

(1) What are the differences between the effect of handwriting and the effect of viewing characters in terms of individuals’ behavioral and ERP responses?
(2) What are the differences between the effect of handwriting and the effect of drawing followed by Chinese recognition in terms of individuals’ behavioral and ERP responses?
(3) What are the differences between the effect of drawing followed by Chinese recognition and the effect of drawing followed by English recognition in terms of behavioral and electroencephalogram (EEG) responses?
(4) What is the difference in lateralization of the facilitative effect of handwriting between children and adults?

## Materials and Methods

### Participants

The University of Science and Technology Beijing (USTB) ethics committee approved the study. Parents of the children and the college students first signed the Informed Consent Form and then completed a background survey of developmental disorders and learning disabilities. After screening, 24 children (15 males, *M*_age_ = 9.5 years, *SD* = 0.86) in grades 3 and 4, who were at the significant period of handwriting development, participated in the experiment. Twenty-four undergraduates (eight males; *M*_age_ = 19.8 years old) from the USTB also participated in the experiment. All the participants were right-handed with normal or corrected-to-normal vision and no history of psychiatric or neurological disorders. Transportation and accommodations were reimbursed for participants who had to travel to the experiment site. The local participants were compensated 30 yuan (approximately $4.50 US) per hour.

### Materials

Study materials included Chinese characters and English words that were selected from the children’s Chinese and English textbooks. These Chinese character materials have been used in previous studies (Guan and Fraundorf, 2020; Guan et al., 2020; Guan and Geva, under review); details about the selection process can be found in Guan et al. (2020). The materials included the prompt, target 1, and target 2. Chinese stimuli included 心， 乙， 人， 飞， 九， 儿； 口， 工， 日， 王， 十， and 田. Characters were selected for target 1 (32 in total) based on the following criteria: (1) high frequency (occur frequently in standard Chinese writing), according to the work of Chen and Shu (2001); (2) easy to embed in complex or compound characters; and (3) simple characters that contained either curved-line strokes or straight-line strokes. Target 2 comprised compound characters that contained the target 1 characters. Target 2 (32 in total) characters were chosen based on configuration (left–right, up–down, inside–outside) and familiarity. The characters-to-be-learned and the targets were counterbalanced with characters’ curving or straight features. The number of strokes for characters of target 2 was always higher than that for the target 1 characters. See Appendix 1 for detailed Chinese stimuli.

The English materials comprised all capital letters or words. During the learning conditions, the stimuli were H, F, I, T, E, L, O, C, Q, and U, six straight-line letters and four curved letters. Target 1 (32 in total) contained 26 capital letters. Target 2 (32 in total) comprised words containing 4–6 of these capital letters. The words chosen were judged to be known to all participants, which controlled for the effect of familiarity. See Appendix 2 for the English stimuli. The judgment task was the same for both Chinese and English: to decide whether target 1 was embedded in target 2.

### Procedures

This study used a within-subject design. The independent variables were four conditions [handwriting Chinese (HC), viewing Chinese (VC), drawing Chinese (DC), and drawing English (DE)]; the dependent variables were behavioral performance [accuracy (ACC) and response time (RT)] and the ERP component (N170).

The experiment used four learning conditions. The first learning condition was VC, under which participants only needed to view the blue stimulus of Chinese words and then respond to the judgment target task by making a binary decision on whether target 2 contained target 1. The second condition was HC, in which participants wrote the blue stimulus of simple Chinese characters on a writing pad and then responded to the same Chinese judgment target task. The third condition was drawing followed by Chinese recognition (DC), which asked participants to draw the priming stimulus (circle, square, triangle, diamond, rectangle, parallel lines, or wavy lines) on the writing pad first and then respond to the Chinese judgment target task. The fourth condition was drawing followed by English recognition (DE), in which participants drew the same priming stimulus as in the DC condition, followed by responding to the English target task.

**FIGURE 1:**
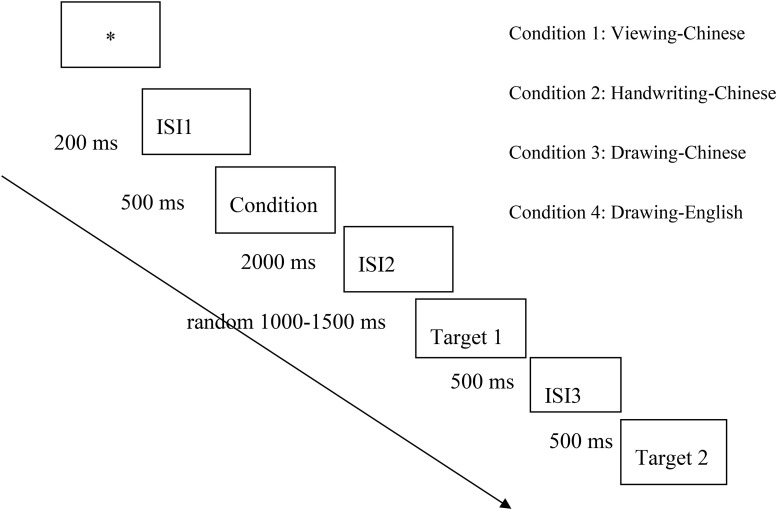
Experimental procedure.

Each participant participated in an EEG test with a total duration of 350 s. The data were collected in the EEG laboratory of the National Institute of Education Science, and all materials appeared in the center of the computer screen. Before the formal experiment, participants participated in a training activity designed to familiarize them with the experimental procedures in all four conditions. See Figure 1 for the flowchart of the presentation. To start, a fixation asterisk appeared on the screen for 200 ms; following the fixation, a blank black screen appeared for 300 ms. Then, there was a 2,000-ms learning phase. In all four conditions, the learning phase began with the stimulus in blue, followed by target 1 in red and then target 2 in white. In the handwriting condition, participants wrote the blue stimulus. In the viewing condition, participants spent the same length of time viewing the stimuli. After a blank black screen appeared for 1,000–1,500 ms (duration chosen at random), the red target 1 was shown to participants for 500 ms followed by a 500-ms blank black screen. Finally, target 2 appeared in white, and participants were instructed to press button “y” if target 2 included target 1 or button “n” if it did not. In a word, participants decided whether target 1 was included in target 2. When participants pressed the button, the screen disappeared; if no button was pressed, the screen remained for 3,500 ms. The program then advanced to the next trial. The EEG recording began upon the onset of the fixation, and continuous EEG recording proceeded, during which the responses to target 1 and target 2 were all marked in the EEG recording.

### Event-Related Potential Data Acquisition and Preprocessing

Response time and accuracy were recorded during ERP data acquisition. ERP data were collected using NeuroScan’s ESI-64 system. Electrode position in this study approximated locations of the international 10–20 system. The study used the left mastoid as the reference electrode. The vertical electrooculogram (VEOG) was recorded using two electrodes placed above and below the midline of the right eye, and the recording electrodes of the horizontal electrooculogram (HEOG) were placed beside the left and right eyes in horizontal alignment with the eyeball.

All electrodes were placed on the scalp using conductive paste to ensure that the impedance of each electrode was kept below 5 KΩ. The EEG data acquisition software was NEUROSCAN. The amplifier was SYNAMPS2, and AC continuous sampling was adopted. Scalp potentials were recorded with a sampling rate of 1,000 Hz, and the bandpass filter is 0.05∼100 Hz.

Offline analysis of EEG data was performed using Curry 7.0. During the recording, the left mastoid was used; later, the data were referenced offline using a reference averaged across the left and right mastoids. First, a constant baseline correction was performed. Second, the data were digitally filtered with a 30-Hz lowpass. Then, the components related to eye movement were removed. In addition, amplitudes exceeding ± 100 μV were also excluded as artifacts. The continuous EEG data were segmented, with the duration of the segmentation starting 200 ms before the onset of target 1 and extending 800 ms after target 1. Finally, the ERP waves were superimposed and averaged, and the baseline correction was performed using the baseline of 200 ms before the stimulus.

### Behavior and Event-Related Potential Data Analyses

For behavioral data, we conducted 4 (learning conditions: VC, HC, DC, and DE) × 2 (children vs. adult as between-subject factor) repeated-measures analyses of variance (ANOVAs) on RT and ACC. For ERP data, according to prior literature (Maurer et al., 2008), the N170 component elicited by Chinese characters has generally been recorded on PO7 and PO8 electrodes, and a lateralization effect has been reported, with the left negative wave larger than the right negative wave (Rossion et al., 2007; Zhang et al., 2011). The stimulus-elicited peak and latency of the N170 at the PO7 and PO8 electrodes of each participant were extracted from the EEG data and analyzed by the statistical models by using SPSS 17.0.4. Here, 4 (learning conditions: VC, HC, DC, and DE) × 2 (electrode position: left PO7 and right PO8) repeated-measures ANOVAs were performed to analyze the amplitude and latency of the N170 of both adults and children. After demonstrating a significant main effect of group and learning condition, as well as their interaction, we broke the analyses down into two groups (children and adults). To answer the first three research questions, we compared three pairs of learning conditions (VC vs. HC, VC vs. DC, DC vs. DE), and to answer the fourth research question regarding the laterality effect, we examined the hemispheric differences in the N170. To correct for multiple comparisons, a Bonferroni correction was applied because the data violated the assumption of sphericity (Bland and Altman, 1995; Chen et al., 2017). A significance level of 0.05 was used for all statistical analyses.

## Results

### Behavioral Results

Because the adults and children were tested using the same materials and had all been trained on the procedures before beginning the trials, behavioral differences between the adults and children can be attributed to their cognitive ability (Palmis et al., 2020). Therefore, behavioral data analysis did not focus on comparisons between adults and child but instead investigated the differences in behavioral performance in the four conditions between groups.

**FIGURE 2:**
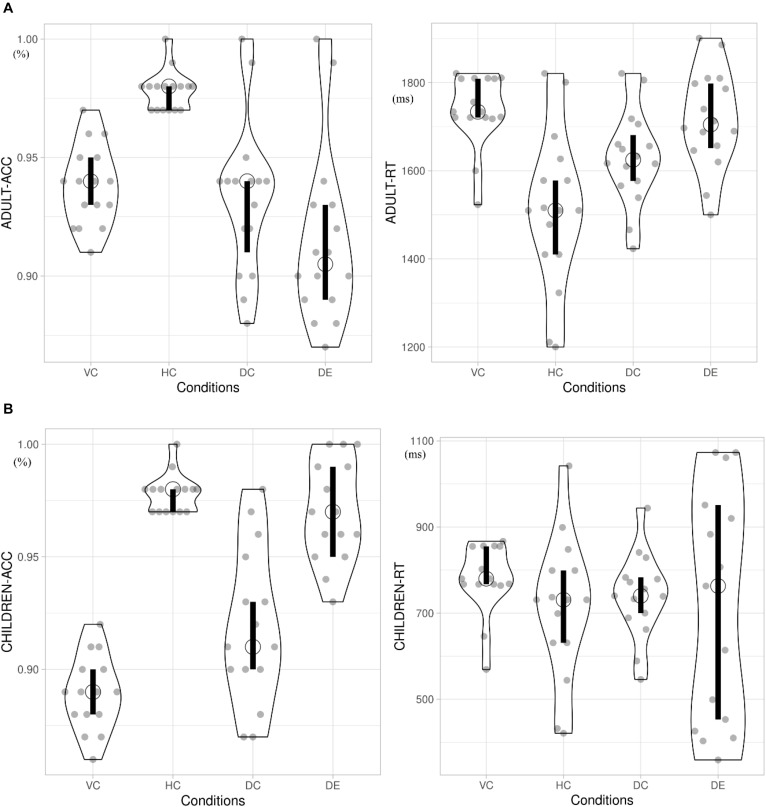
**(A)** Behavioral data of adults. Open circle indicates the median in each condition. Vertical bar indicates the 95% confidence interval for each median determined by bootstrapping. ACC, accuracy; RT, response time; VC, viewing Chinese; HC, handwriting Chinese; DC, drawing followed by Chinese recognition; DE, drawing followed by English recognition. **(B)** Behavioral data of children. Open circle indicates the median in each condition. Vertical bar indicates the 95% confidence interval for each median determined by bootstrapping.

For behavioral data analyses, both ACC and RT for target 2 were collected. The aggregated means per subject per condition were submitted for ACC analyses. RTs were recorded from the onset of target 2 to the button press. Outliers were determined as those RTs located in the extreme 5% on either end of the Z-normalized distribution of RTs. This is equivalent to removing RTs above and below 1.65 SD of each individual participant mean RT. Overall, this resulted in 7.5% of trials being excluded as outliers, within the 5–10% recommended by Ratcliff (1993). Table 1 shows the descriptive statistics of mean and SD of both ACC and RT for each of the four conditions. Figures 2A,B present violin plots summarizing the behavioral data for both children and adults.

**TABLE 1:** Mean and SD of both ACC and RTs in the four conditions.

Condition	RT			ACC		
	Adults	Children	Cohen’s *d*	Adults	Children	Cohen’s *d*
VC	779 (149)	1,734 (282)	1.71	0.88 (0.23)	0.94 (0.29)	1.44
HC	711 (137)	1,578 (261)	1.60	0.98 (0.17)	0.98 (0.27)	3.28
DC	739 (122)	1,628 (259)	1.63	0.90 (0.16)	0.94 (0.35)	0.36
DE	713 (137)	1,708 (301)	1.51	0.97 (0.19)	0.91 (0.33)	1.17

*ACC, accuracy; RT, response time; VC, viewing Chinese; HC, handwriting Chinese; DC, drawing followed by Chinese recognition; DE, drawing followed by English recognition. Standard deviation of each measure per condition presented in parentheses. We calculated Cohen’s *d* by using the following formula: [4η^2^/1-η^2^]^1/2^. Cohen’s *d* < 0.2 indicates a small effect size, 0.2 < Cohen’s *d* < 0.8 indicates a medium effect size, and Cohen’s *d* > 0.8 indicates a large effect size (Fritz et al., 2012).*

Four repeated-measures ANOVAs were performed using a single factor (learning conditions: VC, HC, DC, and DE) by submitting RT and ACC for each condition across children and adult groups. The group (child vs. adult) factor was used as the between-participant factor. RT and ACC of children and adults demonstrated significant effects of learning condition. For RT, there was a significant effect of learning condition [*F*(3,84) = 6.910, *p* = 0.003, η^2^ = 0.198] and condition × group interaction [*F*(3,84) = 4.297, *p* = 0.007, η^2^ = 0.133]. For ACC, there was a significant effect of learning condition [*F*(3,84) = 64.539, *p* < 0.001, η^2^ = 0.697] and a significant condition × group interaction [*F*(3,84) = 29.951, *p* < 0.001, η^2^ = 0.517]. Therefore, three sets of *post hoc* analyses were carried out below in children and adults, respectively.

#### Comparing Handwriting vs. Viewing

Among children, the RT in HC (*M* = 1,578 ms) was significantly shorter than that in VC (1,734 ms) [*F*(1,15) = 2.047, *p* < 0.001, η^2^ = 0.68], and the ACC rate in HC (*M* = 0.98) was significantly higher than that in VC (*M* = 0.94) [*F*(1,15) = 334.657, *p* < 0.001, η^2^ = 0.923]. For adults, the patterns were the same. Their RT in HC (*M* = 711 ms) was significantly shorter than that in VC (*M* = 779 ms) [*F*(1,15) = 21.87, *p* < 0.001, η^2^ = 0.422], and ACC of HC (*M* = 0.98) was significantly higher than that of VC (*M* = 0.88) [*F*(1,15) = 72.624, *p* < 0.001, η^2^ = 0.708].

#### Comparing Handwriting vs. Drawing Followed by Chinese Recognition

For children, the RT in HC (*M* = 1,734 ms) was significantly longer than that in DC (*M* = 1,628 ms) [*F*(1,15) = 0.328, *p* < 0.001, η^2^ = 0.012], and the ACC in HC (*M* = 0.98) was significantly higher than that in DC (*M* = 0.94) [*F*(1,15) = 41.502, *p* < 0.001, η^2^ = 0.597]. For adults, there was a significantly longer RT of HC (*M* = 779 ms) compared with that of DC (*M* = 739 ms) [*F*(1,15) = 5.278, *p* = 0.029, η^2^ = 0.15], and ACC in HC (*M* = 0.98) was significantly higher than that in DC (*M* = 0.90) [*F*(1,15) = 30.198, *p* < 0.001, η^2^ = 0.502].

#### Comparing Drawing Followed by Chinese Recognition vs. Drawing Followed by English Recognition

For children, the RT of Chinese recognition in the DC condition (*M* = 1,628 ms) was not significantly different from that of English recognition in the DE condition (*M* = 1,708) [*F*(1,15) = 0.132, *p* = 0.719, η*^2^* = 0.005], and the ACC of DC (*M* = 0.98) was higher than that of the DE condition (*M* = 0.91) [*F*(1,15) = 23.083, *p* < 0.001, η*^2^* = 0.452]. For adults, there was no difference between ACC [*F*(1,15) = 2.047, *p* = 0.16, η*^2^* = 0.06] and no difference in RT [*F*(1,15) = 5.278, *p* = 0.08, η^2^ = 0.15] in the two drawing conditions.

### Event-Related Potential Results

**FIGURE 3:**
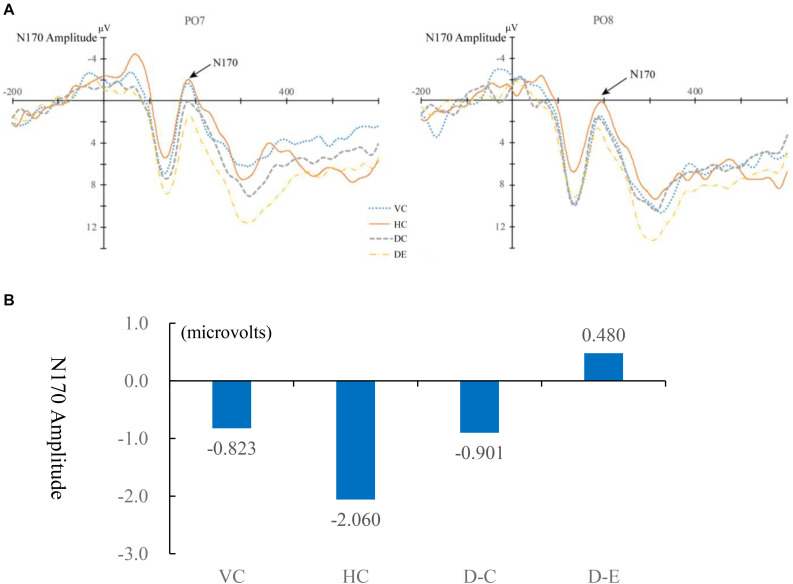
**(A)** Event-related potential (ERP) waveforms of the N170 under four conditions for children for the left (PO7) and right (PO8) parietal leads. VC, viewing Chinese; HC, handwriting Chinese; DC, drawing followed by Chinese recognition; DE, drawing followed by English recognition. **(B)** Differences between the four conditions for children in the amplitude of N170.

**FIGURE 4:**
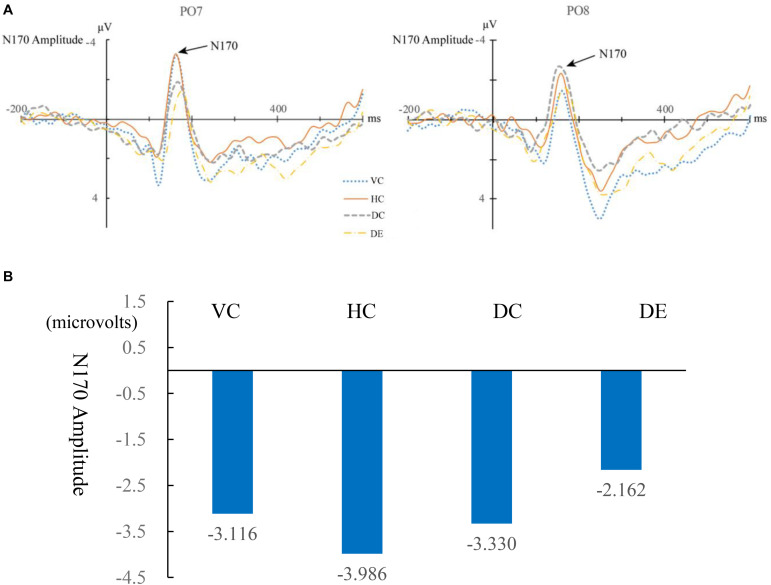
**(A)** Event-related potential (ERP) waveforms of N170 under four conditions for adults for the left (PO7) and right (PO8) parietal leads. VC, viewing Chinese; HC, handwriting Chinese; DC, drawing followed by Chinese recognition; DE, drawing followed by English recognition. **(B)** Differences between the four conditions for adults in the amplitude of N170.

**FIGURE 5:**
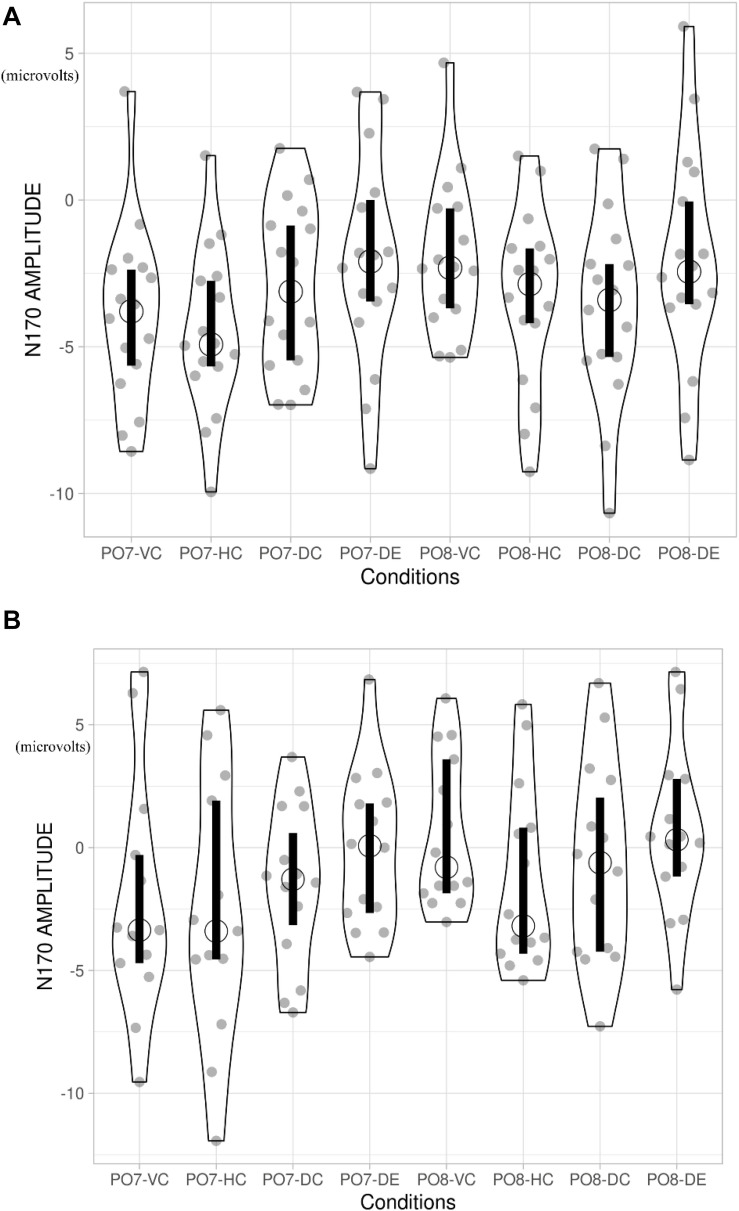
**(A)** N170 amplitude of adults. Open circle indicates the median of the data. Vertical bar indicates the 95% confidence interval for each median determined by bootstrapping. VC, viewing Chinese; HC, handwriting Chinese; DC, drawing followed by Chinese recognition; DE, drawing followed by English recognition. **(B)** N170 amplitude of children. Open circle indicates the median of the data. Vertical bar indicates the 95% confidence interval for each median determined by bootstrapping.

The original ERP waveforms that marked target 1 responses at PO7 and PO8 for children are shown in Figure 3A and for adults are shown in Figure 4A. A 4 (learning conditions: VC, HC, DC, and DE) × 2 (hemisphere: left PO7 and right PO8) × 2 (group: adult vs. children) repeated-measures ANOVA was carried out on the N170 amplitude. The results revealed a significant main effect of condition [*F*(3,81) = 5.536, *p* = 0.002, η^2^ = 0.165], main effect of group [*F*(1,28) = 5.344, *p* = 0.07, η*^2^* = 0.177], and significant condition × hemisphere × group interaction [*F*(3,81) = 0.954, *p* = 0.419, η^2^ = 0.02]. Moreover, we observed a significant two-way interaction of condition × hemisphere [*F*(3,81) = 6.858, *p* = 0.000, η^2^ = 0.197] and a significant two-way interaction of group × hemisphere [*F*(3,81) = 5.183, *p* < 0.001, η^2^ = 0.152], showing that there is a different pattern across hemispheres among the four conditions and between children and adults. Therefore, we broke down the N170 amplitude analyses in a condition comparison within children and adult groups separately. Table 2 shows the descriptive statistics of EEG data of all conditions. In addition, like previous studies (Maurer et al., 2008; Yum et al., 2014; Yum and Law, 2021), latency was analyzed, but the results were not significant, so we only report the EEG amplitude data results. Differences between the conditions are shown for children in Figure 3B and for adults in Figure 4B. Table 3 shows the correlation matrix for behavioral and EEG data. Figures 5A,B present summaries of the N170 amplitude data for both children and adults.

**TABLE 2:** Mean (SD) ERP magnitude at PO7 and PO8 for four conditions.

	Adults		Children	
	PO7	PO8	PO7	PO8
VC	–3.95 (2.96)	–1.98 (2.60)	–2.16 (4.67)	0.57 (2.96)
HC	–4.49 (2.74)	–3.37 (2.94)	–2.69 (5.06)	–1.35 (3.62)
DC	–2.99 (2.80)	–3.62 (3.22)	–1.54 (3.15)	–0.62 (3.96)
DE	–2.16 (3.46)	–2.07 (3.69)	–0.07 (3.08)	0.57 (3.38)

*ERP, event-related potential; VC, viewing Chinese; HC, handwriting Chinese; DC, drawing followed by Chinese recognition; DE, drawing followed by English recognition. Standard deviation of each measure per condition is presented in parentheses.*

**TABLE 3:** Correlational matrix between mean amplitude of P07 P08 and behavioral responses of RT and ACC.

		Adult								Children							
		RT				ACC				RT				ACC			
		VC	HC	DC	DE	VC	HC	DC	DE	VC	HC	DC	DE	VC	HC	DC	DE
PO7	VC	0.263** (0.023)				–0.235* (0.008)				0.198 (0.177)				–0.210 (0.164)			
	HC		0.103 (0.254)				–0.302 * (0.048)				0.348 (0.052)				–0.294 (0.183)		
	DC			0.122 (0.103)				0.059 (0.829)				0.151 (0.386)				–0.380** (0.007)	
	DE				0.149 (0.581)				0.087 (0.748)				–0.120 (0.135)				0.196 (0.501)
PO8	VC	0.170 (0.158)				–0.270* (0.021)				–0.257 (0.376)				0.087 (0.767)			
	HC		0.208 (0.440)				–0.298 (0.127)				0.139 (0.116)				–0.048 (0.870)		
	DC			0.176 (0.301)				0.188 (0.485)				0.003 (0.993)				0.182 (0.533)	
	DE				0.208 (0.439)				–0.116 (0.668)				0.163 (0.578)				0.081 (0.784)

*p < 0.05, **p < 0.01; Significance levels are presented in paratheses.

#### Comparing Handwriting vs. Viewing

For children, there was a greater N170 amplitude during HC than that during VC [*F*(1,15) = 0.72, *p* = 0.035, η*^2^* = 0.03], showing that handwriting facilitates recognition of Chinese characters. For adults, the patterns were the same. The amplitude of the N170 was significantly greater for HC than for VC [*F*(1,15) = 1.879, *p* = 0.029, η*^2^* = 0.059].

#### Comparing Handwriting vs. Drawing Followed by Chinese Recognition

For both children and adults, there was no difference in N170 amplitude for HC and DC [*F*(1,15) = 2.191, *p* > 0.05, η*^2^* = 0.068 for adults; *F*(1,15) = 0.473, *p* > 0.05, η*^2^* = 0.019 for children].

#### Comparing Drawing Followed by Chinese Recognition vs. Drawing Followed by English Recognition

For children, DC elicited a significantly larger N170 response than DE [*F*(1,15) = 15.07, *p* = 0.02, η*^2^* = 0.53]. For adults, the N170 amplitude was also greater for DC than DE [*F*(1,15) = 0.527, *p* = 0.04, η*^2^* = 0.017].

#### Laterality Effect

**FIGURE 6:**
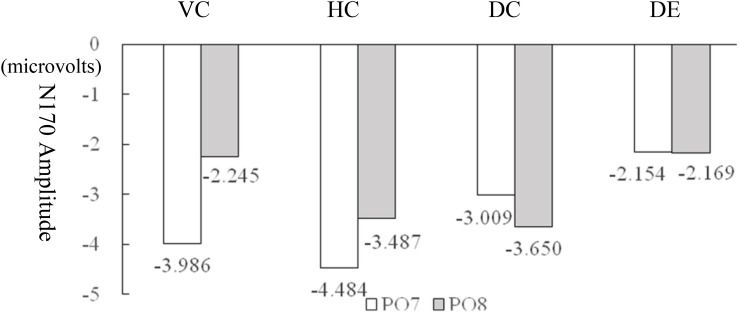
Lateralization effect for adults in the N170 amplitude under the four conditions. VC, viewing Chinese; HC, handwriting Chinese; DC, drawing followed by Chinese recognition; DE, drawing followed by English recognition.

For adults, the peak value of N170 of the left hemisphere PO7 was significantly higher than that of the right hemisphere PO8 for HC [*F*(1,16) = 7.794, *p* = 0.013, η^2^ = 0.328], VC [*F*(1,16) = 9.208, *p* = 0.005, η^2^ = 0.365], but the laterality effects were not significant in the two drawing conditions [DC: *F*(1,16) = 0.327, *p* = 0.572, η^2^ = 0.011; DE: *F*(1,16) = 0.004, *p* = 0.948, η^2^ = 1.461e?-4]. For children, the four conditions showed no significant laterality [VC: *F*(1,14) = 3.083, *p* = 0.091, η^2^ = 0.110; HC: *F*(1,14) = 0.585, *p* = 0.452, η^2^ = 0.023; DC: *F*(1,14) = 0.428, *p* = 0.519, η^2^ = 0.016; DE: *F*(1,14) = 3.083, *p* = 0.091, η^2^ = 0.110]. Figure 6 shows the lateralization of the N170 for the four conditions. Please see Table 4 for a summary of the behavioral and N170 results.

**TABLE 4:** Summary table of behavioral and EEG results.

		HC vs. VC	HC vs. DC	DC vs. DE	
Adults	ACC	> (1.46)	> (1.23)	>(1.16)	
	RT	<(1.13)	>(0.67)	ns	
	N170	>(0.16)	ns	>(0.22)	
		HC	VC	DC	DE
	Laterality	L > R (0.99)	L > R (1.05)	ns	ns
Children	ACC	>(1.66)	>(1.34)	ns	
	RT	<(0.61)	>(0.19)	ns	
	N170	>(0.09)	ns	>(1.26)	
		HC	VC	DC	DE
	Laterality	ns	ns	ns	ns

*Effect sizes represented by Cohen’s *d* for the group comparison are reported in the parentheses. We calculated Cohen’s *d* using the following formula: [4η^2^/1-η^2^]^1/2^. Cohen’s *d* < 0.2 indicates a small effect size, 0.2 < Cohen’s *d* < 0.8 indicates a medium effect size, and Cohen’s *d* > 0.8 indicates a large effect size (Fritz et al., 2012). EEG, electroencephalogram; VC, viewing Chinese; HC, handwriting Chinese; DC, drawing followed by Chinese recognition; DE, drawing followed by English recognition; ACC, rates for the binary decision; RT, response time; L, left hemisphere; R, right hemisphere.*

## Discussion

We compared HC with VC characters and two other drawing conditions, i.e., drawing shapes followed by Chinese recognition (DC) and drawing shapes followed by English recognition (DE). There were four main findings. First, we revealed a facilitating effect, for both adults and children, of HC on behavior and the N170 compared to VC. Second, we revealed a facilitating effect on ACC of HC on behavior measures compared to drawing shapes. Although we did not find neural effects, handwriting appears to enhance visual word recognition more than simply drawing shapes. Third, we found that drawing shapes appeared to have a larger effect on the N170 of Chinese characters compared to English words. Finally, we found a left lateralization of the effect of HC and VC, suggesting greater specialization in adults compared to children.

The facilitating effect on HC is represented by its comparison with VC, with shorter RTs and higher ACC in HC compared to VC. The peak of the N170 for HC was also significantly larger than that of VC. This ERP finding suggests that, in comparison to VC, HC enhanced the processing of Chinese characters for both adults and children. The finding that a stronger N170 was triggered by the HC than the VC condition suggests that the N170 indicates enhanced orthographic word recognition. This finding is consistent with the results of Liu and Perfetti (2003), who found this pattern for Chinese–English bilinguals, and with a series of handwriting training studies (Guan et al., 2011, 2015, 2020; Guan and Fraundorf, 2020). Handwriting training appears to enhance familiarity with the orthographic representation of the word. This finding is also consistent with a study with artificial orthographies by Yoncheva et al. (2010), who found that the unit size acquired through training influences N170 response to visual words, which was greater when training was based on the small unit size (i.e., grapheme compared to whole word). For both children and adults in our study, handwriting training drew more attention to the small units within the word form. The judgment task asked them to decide whether a simpler character was embedded in the more complicated whole character. Paying attention to the local features may enhance the early processing of Chinese characters, thus affecting the N170.

Handwriting practice likely increases motor–sensory integration to facilitate visual recognition by focusing on the detailed visual–orthographic components of stroke composition (Guan et al., 2011). Guan et al. (2015) found that the improvement of handwriting quality predicted gains in reading comprehension when previous knowledge was controlled for. Handwriting has a sensory–motor source for native language, forming a mental model accompanied by a new neural motor memory (Shadmehr and Holcomb, 1997). Sensory–motor training facilitates language cognition (Guan and Wang, 2017). That is, people who can better understand the visual–motor coupling in this language are usually those who more effectively learn the visual–orthographic representation of the written language.

The higher ACC level for HC than DC revealed that HC characters led to better performance than drawing followed by Chinese recognition, suggesting that handwriting helps to coordinate the brain, eyes, and fingers to establish a subtle representation for sub-lexical word forms (Guan et al., 2011). Handwriting may accelerate the perception of Chinese characters for both adults and children (Guan et al., 2015). However, the reaction times for DC were faster than those for HC for both adults and children, and the EEG results for HC and DC were not significantly different. These mixed results suggest that the N170 may be influenced by both handwriting and drawing.

The different performance in DE and DC may possibly reflect differences in the ways adults and children process Chinese and English. Foremost, our results comparing between DC and DE may just reflect the language difference effect itself. Processing of Chinese may involve a category-specific form of processing. Indeed, a larger N170 has been observed for Chinese characters relative to English, along with a more left-lateralized N170 for Chinese characters for English–Chinese bilinguals compared to English-only participants (Wong et al., 2005). Therefore, the processing of Chinese may, like faces, involve “special” processing in the brain, although the hemispheric lateralization of the N170 to such stimuli is still unclear.

Meanwhile, there was an enhancement of the N170 in drawing followed by Chinese recognition (DC) compared with drawing followed by English recognition (DE), probably reflecting a native language effect. Most children in China only begin to learn English in the third grade. In our study, Chinese was the native language for all participants, and therefore, they were much more familiar with Chinese characters than English letters. Our finding that native Chinese-speaking adults and children displayed a greater N170 effect on Chinese than their second language (English) is consistent with findings of Liu and Perfetti (2003) that the N170 perceptual effect of a native language was greater than that of a second language. Research has shown that the N170 indexes visual–orthographic processing. Orthographic stimuli (such as words, pseudo-words, and consonant strings) produced greater N170 effects than non-orthographic stimuli (such as symbols) (Bentin et al., 1999; Pylkkanen and Marantz, 2003; Simon et al., 2004). Chinese adults and children are much more familiar with Chinese than English, which may have produced a larger N170 component.

Adults showed a lateralization of the N170 effect in the HC and VC conditions, but the children did not. Adults have developed much experience with written language; therefore, they show N170 lateralization during the viewing and handwriting conditions. People are not born with N170 lateralization nor does it exist in early cognition in children. Rather, it is the result of humans’ experience with written language in their later years. This pattern of results is in line with the existing literature that has found a left-lateralized effect of the N170 for Chinese characters (Maurer et al., 2008). Previous studies have reported an enhanced N170 for words in syllabic writing systems compared to control stimuli but did not explicitly test left lateralization (Kim et al., 2004; Shirahama et al., 2004). In addition, left lateralization has been shown to be characteristic of visual expertise for words written in alphabetic scripts (Bentin et al., 1999; Rossion et al., 2003; Maurer et al., 2005b). The current results suggest that similar processes underlie the left-lateralized N170 in logographic writing systems and writing systems that associate characters with larger phonological units, such as syllables.

Remarkably, Cao et al. (2011) tested all four age groups (7-, 9-, and 11-year-olds, as well as college students); even the youngest group showed a left-lateralized N170 response for Chinese characters, suggesting that a relatively specialized mechanism for processing Chinese characters is already emergent by as early as 7 years of age. However, our results showed that adults demonstrated laterality, while children (*M*_age_ = 9.5 years) did not. Visual form familiarity serves as an important driver for the increased and left-lateralized N170 response among adults. Xue et al. (2019) found an increased and left-lateralized N170 response for regular characters compared to cursive characters that were less familiar. It is possible that the amount of training was not sufficient for increasing the familiarity of the visual characters for the children in our study.

Our study is not without limitation. Because we used the same stimuli across groups, the difficulty level of our stimuli was not the same in children and adults. Future research should consider the varying difficulty levels across ages. In addition, because the participants only engaged in handwriting or drawing for a few seconds, the modest effects might be due to the shorter duration. Longer exposure to the learning conditions might lead to greater effect sizes. Children might benefit from longer handwriting experiences in those conditions. In addition, handwriting curved letters in comparison to the straight-line letters/characters might have different effects on the brain’s visual-form areas (Ose Askvik et al., 2020). Finally, more fine-grained examination of the EEG before 170-ms post stimulus onset might also be considered (Woodman, 2010), as this might reveal an effect of handwriting on sensory processing (Pratt, 2011), word recognition (Hillyard et al., 1998), or visual discrimination (Vogel and Luck, 2000).

## Conclusion

We found that HC produced a larger N170 and better performance than VC and better performance than drawing shapes for both children and adults. The key mechanism under these effects may be visual–motor integration. The interaction between visual and motor areas may enhance orthographic representations. The left lateralization of the N170 effect was seen in adults and not children, suggesting that greater familiarity with characters and more practice with handwriting are necessary to improve the quality of the orthographic representations in children. Future studies should further explore different methods to facilitate orthographic perception through handwriting.

## Data Availability Statement

The raw data supporting the conclusions of this article will be made available by the authors, without undue reservation.

## Ethics Statement

The studies involving human participants were reviewed and approved by the University of Science and Technology Beijing (USTB).

## Author Contributions

CG andWMdesigned the study, collected and analyzed the data, wrote the manuscript. ES and JB provided the comments and proofed the manuscript. All authors contributed to the article and approved the submitted version.

## Conflict of Interest

The authors declare that the research was conducted in the absence of any commercial or financial relationships that could be construed as a potential conflict of interest.
