# Supplementary material for: Effect of Handwriting on Visual Word Recognition in Chinese Bilingual Children and Adults
Source: Front Psychol. 2021 May 28;12:628160. doi: 10.3389/fpsyg.2021.628160 (PMC8194694; doi:10.3389/fpsyg.2021.628160)
Supplement: Supplementary file 1 [file Data_Sheet_1.docx]

Appendix 1

Chinese stimuli

| Condition | Trial Number | Prompt | Target 1 | Target 2 | Embedded |
| --- | --- | --- | --- | --- | --- |
| 1 | 1 | 心 | 也 | 地_(6)_ | 1 |
| 1 | 2 | 乙 | 元 | 玩_(8)_ | 1 |
| 1 | 3 | 工 | 击 | 约_(6)_ | 2 |
| 1 | 4 | 日 | 卡 | 住_(7)_ | 2 |
| 1 | 5 | 九 | 乙 | 旯_(6)_ | 2 |
| 1 | 6 | 飞 | 人 | 液_(11)_ | 2 |
| 1 | 7 | 口 | 田 | 佃_(7)_ | 1 |
| 1 | 8 | 工 | 甘 | 甜_(11)_ | 1 |
| 1 | 9 | 人 | 包 | 抱_(8)_ | 1 |
| 1 | 10 | 人 | 乙 | 亿_(3)_ | 1 |
| 1 | 11 | 日 | 占 | 冲_(6)_ | 2 |
| 1 | 12 | 口 | 土 | 骂_(9)_ | 2 |
| 1 | 13 | 日 | 立 | 泣_(8)_ | 1 |
| 1 | 14 | 王 | 五 | 伍_(6)_ | 1 |
| 1 | 15 | 儿 | 兀 | 突_(9)_ | 2 |
| 1 | 16 | 儿 | 北 | 务_(5)_ | 2 |
| 1 | 17 | 飞 | 丈 | 杖_(7)_ | 1 |
| 1 | 18 | 心 | 凡 | 梵_(11)_ | 1 |
| 1 | 19 | 工 | 巨 | 曹_(11)_ | 2 |
| 1 | 20 | 王 | 平 | 沮_(8)_ | 2 |
| 1 | 21 | 十 | 日 | 早_(6)_ | 1 |
| 1 | 22 | 十 | 工 | 贡_(7)_ | 1 |
| 1 | 23 | 心 | 叉 | 序_(7)_ | 2 |
| 1 | 24 | 儿 | 己 | 幽_(9)_ | 2 |
| 2 | 1 | 乙 | 几 | 秃_(7)_ | 1 |
| 2 | 2 | 飞 | 七 | 斧_(8)_ | 1 |
| 2 | 3 | 十 | 王 | 园_(7)_ | 2 |
| 2 | 4 | 田 | 十 | 梧_(11)_ | 2 |
| 2 | 5 | 田 | 口 | 杏_(7)_ | 1 |
| 2 | 6 | 口 | 三 | 闫_(6)_ | 1 |
| 2 | 7 | 九 | 儿 | 厄_(4)_ | 2 |
| 2 | 8 | 九 | 大 | 网_(6)_ | 2 |
| 2 | 9 | 九 | 冬 | 疼_(10)_ | 1 |
| 2 | 10 | 乙 | 力 | 历_(4)_ | 1 |
| 2 | 11 | 田 | 耳 | 杖_(7)_ | 2 |
| 2 | 12 | 十 | 二 | 伶_(7)_ | 2 |
| 2 | 13 | 田 | 干 | 赶_(10)_ | 1 |
| 2 | 14 | 王 | 由 | 迪_(8)_ | 1 |
| 2 | 15 | 儿 | 飞 | 或_(8)_ | 2 |
| 2 | 16 | 乙 | 允 | 父_(4)_ | 2 |
| 2 | 17 | 飞 | 么 | 麽_(14)_ | 1 |
| 2 | 18 | 心 | 幺 | 系_(7)_ | 1 |
| 2 | 19 | 口 | 非 | 卞_(4)_ | 2 |
| 2 | 20 | 王 | 中 | 亚_(6)_ | 2 |
| 2 | 21 | 工 | 甲 | 果_(8)_ | 1 |
| 2 | 22 | 日 | 巾 | 布_(7)_ | 1 |
| 2 | 23 | 人 | 个 | 书_(4)_ | 2 |
| 2 | 24 | 人 | 井 | 隶_(8)_ | 2 |
| 3 | 1 | 工 | 击 | 陆_(7)_ | 1 |
| 3 | 2 | 日 | 卡 | 咔_(8)_ | 1 |
| 3 | 3 | 心 | 也 | 化_(4)_ | 2 |
| 3 | 4 | 人 | 乙 | 乳_(8)_ | 2 |
| 3 | 5 | 口 | 田 | 针_(7)_ | 2 |
| 3 | 6 | 工 | 甘 | 忻_(7)_ | 2 |
| 3 | 7 | 乙 | 元 | 阮_(6)_ | 1 |
| 3 | 8 | 飞 | 人 | 纵_(7)_ | 1 |
| 3 | 9 | 日 | 占 | 钻_(10)_ | 1 |
| 3 | 10 | 口 | 土 | 垚_(9)_ | 1 |
| 3 | 11 | 乙 | 九 | 忿_(8)_ | 2 |
| 3 | 12 | 儿 | 兀 | 多_(6)_ | 2 |
| 3 | 13 | 飞 | 丈 | 仗_(7)_ | 1 |
| 3 | 14 | 儿 | 北 | 背_(9)_ | 1 |
| 3 | 15 | 日 | 立 | 机_(6)_ | 2 |
| 3 | 16 | 王 | 五 | 仨_(5)_ | 2 |
| 3 | 17 | 工 | 巨 | 苣_(7)_ | 1 |
| 3 | 18 | 王 | 平 | 苹_(8)_ | 1 |
| 3 | 19 | 乙 | 几 | 芬_(7)_ | 2 |
| 3 | 20 | 九 | 冬 | 疥_(9)_ | 2 |
| 3 | 21 | 心 | 凡 | 芃_(6)_ | 1 |
| 3 | 22 | 飞 | 七 | 皂_(7)_ | 1 |
| 3 | 23 | 田 | 口 | 肯_(8)_ | 2 |
| 3 | 24 | 口 | 三 | 闭_(6)_ | 2 |

**Note**: Condition: (1-viewing 2-hearing 3- drawing followed by Chinese recognition). Embedded: 1- refers to target 1 embedded in target 2. 2 - refers to target 1 not embedded in target 2. stroke number of Target 2 is presented in the bottom right bracket.

Appendix 2

English stimuli

| Condition | Trial Number | Prompt | Target 1 | Target 2 | Embedded |
| --- | --- | --- | --- | --- | --- |
| 4 | 1 | －(horizontal line) | O | ORANGE | 1 |
| 4 | 2 | (rising line) | L | HOPE | 2 |
| 4 | 3 | (moon shape) | F | MANGO | 2 |
| 4 | 4 | ≈ (approximate equal) | X | SIXTY | 1 |
| 4 | 5 | X (cross shape) | G | PEAR | 2 |
| 4 | 6 | (moon shape) | A | TEACH | 1 |
| 4 | 7 | (moon shape) | W | SWEAR | 1 |
| 4 | 8 | (mountain shape) | M | MOTHER | 1 |
| 4 | 9 | X (cross shape) | V | VICTOR | 1 |
| 4 | 10 | (rising line) | M | SNOW | 2 |
| 4 | 11 | ≈ (approximate equal) | I | DANCE | 2 |
| 4 | 12 | (semicircle shape) | X | ZEROX | 1 |
| 4 | 13 | (rising line) | A | WALK | 1 |
| 4 | 14 | －(horizontal line) | C | COLOR | 1 |
| 4 | 15 | ≈ (approximate equal) | L | HIGHT | 2 |
| 4 | 16 | (semicircle shape) | B | CURVE | 2 |
| 4 | 17 | X (cross shape) | R | CHART | 1 |
| 4 | 18 | －(horizontal line) | K | ROBOT | 2 |
| 4 | 19 | (moon shape) | V | HAPPY | 2 |
| 4 | 20 | ❤ (heart shape) | S | OUST | 1 |
| 4 | 21 | －(horizontal line) | E | DULL | 2 |
| 4 | 22 | X(cross shape) | R | HAIR | 1 |
| 4 | 23 | (moon shape) | V | PAVE | 1 |
| 4 | 24 | ≈ (approximate equal) | P | BLUE | 2 |
| 4 | 25 | －(horizontal line) | W | GOLD | 2 |
| 4 | 26 | X (cross shape) | M | SMOG | 1 |
| 4 | 27 | ≈ (approximate equal) | P | FINGER | 2 |
| 4 | 28 | (moon shape) | O | PAGE | 2 |
| 4 | 29 | (semicircle shape) | F | WAFFLE | 1 |
| 4 | 30 | ≈ (approximate equal) | S | PAPER | 2 |
| 4 | 31 | X (cross shape) | Z | DRAFT | 2 |
| 4 | 32 | (rising line) | D | EDGE | 1 |
| 4 | 33 | －(horizontal line) | T | TIRED | 1 |
| 4 | 34 | ≈ (approximate equal) | J | HITE | 2 |
| 4 | 35 | (moon shape) | S | ERASER | 1 |
| 4 | 36 | ❤ (heart shape) | R | ROCK | 1 |
| 4 | 37 | －(horizontal line) | S | SLEEP | 1 |
| 4 | 38 | X (cross shape) | E | PIGGY | 2 |
| 4 | 39 | (moon shape) | C | CLAW | 1 |
| 4 | 40 | ≈ (approximate equal) | O | BREAK | 2 |
| 4 | 41 | (rising line) | C | DARK | 2 |
| 4 | 42 | (moon shape) | J | PHONE | 2 |
| 4 | 43 | X (cross shape) | O | LIQUE | 2 |
| 4 | 44 | －(horizontal line) | F | SWIFT | 1 |
| 4 | 45 | ≈ (approximate equal) | D | EDITER | 1 |
| 4 | 46 | (semicircle shape) | G | SONG | 1 |
| 4 | 47 | －(horizontal line) | D | GRASP | 2 |
| 4 | 48 | (mountain shape) | Y | PILLOW | 2 |

**Note**. Condition 4: the DE condition= drawing followed by English recognition. Embedded: 1- refers to target 1 embedded in target 2. 2 - refers to target 1 not embedded in target 2
